# Description of vital signs data measurement frequency in a medical/surgical unit at a community hospital in United States

**DOI:** 10.1016/j.dib.2017.11.053

**Published:** 2017-11-21

**Authors:** Erina Ghosh, Larry Eshelman, Lin Yang, Eric Carlson, Bill Lord

**Affiliations:** Philips Research North America, United States

## Abstract

Vitals signs are measured at scheduled intervals by nurses in typical general wards. Vital signs may be measured more frequently if the patient condition deteriorates. In many units, the vital signs measurement frequency for some patients is different from the scheduled frequency due to various reasons such as staffing, patient acuity etc. In this article, we describe the actual measurement frequency in patients admitted to general ward in a community hospital in Arizona, US. We present the data in the form of 2 sets of graphs. The first set of graphs are histograms which show the distribution of the number of measurements in a 24 h period for 6 different vital signs. The second set of graphs show the proportion of the patient population who had a measurement of a vital sign for each hour of the last day of patient's general ward stay. The significance of this data on predicting deterioration is discussed in Ghosh et al. (2017) [1].

**Specifications Table**

**Table:** 

Subject area	*Medicine*
More specific subject area	*Clinical Practice in General Ward*
Type of data	*Graph*
How data was acquired	*Electronic Medical Records*
Data format	*Analyzed*
Experimental factors	*The data was anonymized and time stamps shifted to prevent identification of the subjects.*
Experimental features	*Patients admitted to medical/surgical units have vital signs measured at scheduled times. Additional measurements are done if necessary. The article describes the typical measurement frequency for patients who were stable and those who deteriorated and were transferred to higher level of care.*
Data source location	*Phoenix, Arizona, USA*
Data accessibility	*Not accessible*

**Value of the data**

• Based on actual general ward data measurements done on a large number of patients (~11 thousand).
• Can serve as a benchmark for comparing data measurement frequencies from other hospitals.
• Can provide insights into how measurement frequencies vary with patient status and ideas for further research in this area.

## 1 Data

**Fig. 1:**
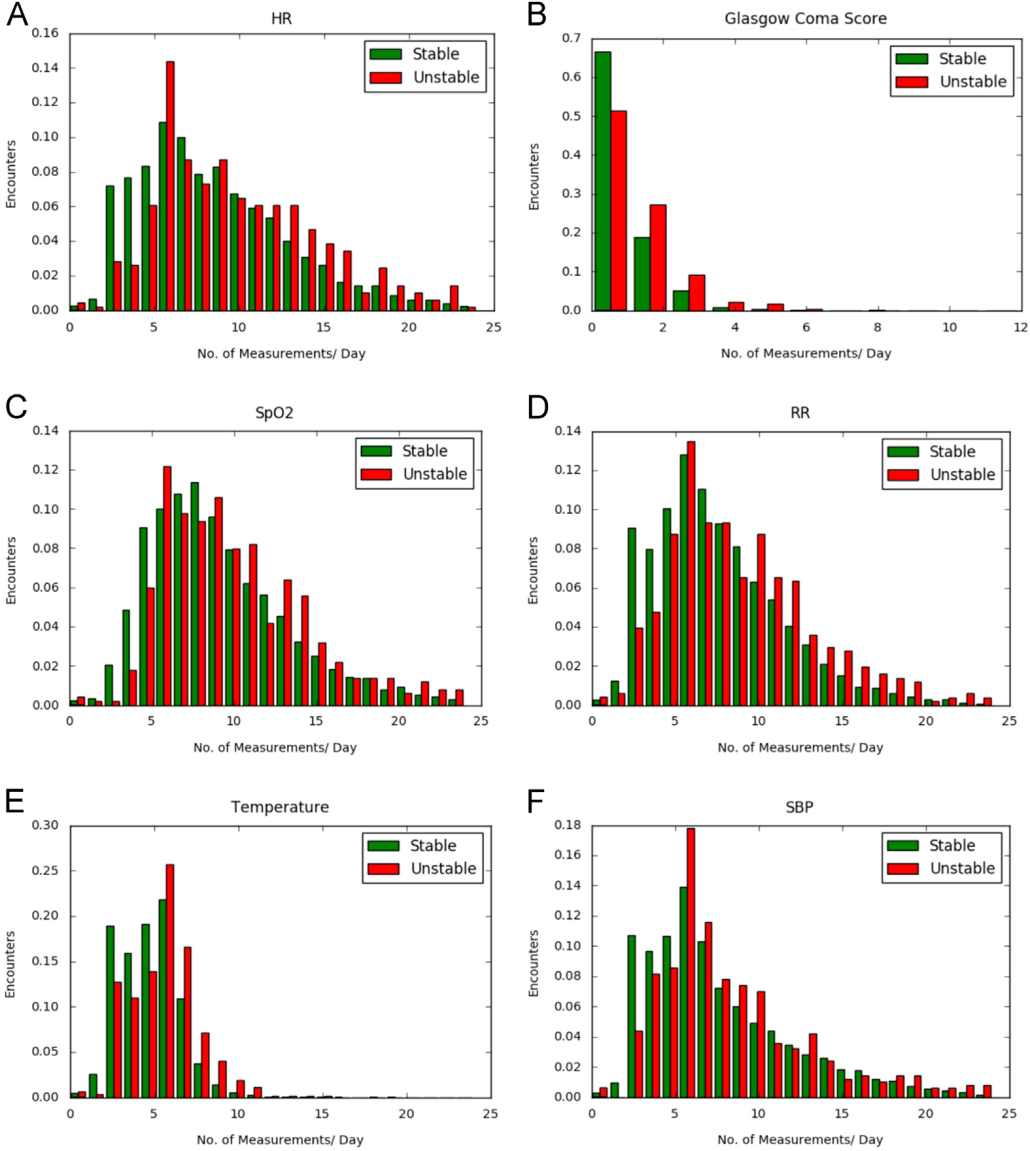
Normalized histogram of number of measurements done per day (24 h period) of general ward stay. Encounters longer than 24 h were selected for this analysis. (A) heart rate, (B) Glasgow Coma Score (for level of consciousness), (C) oxygen saturation, (D) respiration rate, (E) temperature and (F) systolic blood pressure. Green bars indicate stable cohort and red bars indicate unstable cohort. Histograms are truncated to 24 measurements per day except Glasgow Coma Score which was truncated to 12 h per day.

Vital signs in this unit are measured by nursing assistants and level of consciousness (LOC) is charted by registered nurses. We analyzed the extracted data to assess data measurement frequency in stable and unstable encounters. Our analysis revealed that vital signs– heart rate (HR), systolic blood pressure ( SBP), respiration rate (RR), temperature were most commonly measured 6 times/day (approximately once every 4 h). LOC was most commonly measured once per day. Pulse oxygen saturation (SpO_2_) was typically measured 8 times/day in the stable cohort and 6 times per day in the unstable cohort. Although the data measurement frequency is similar in the stable & unstable cohorts, analysis revealed that unstable encounters were more likely to have higher number of measurements (>15/day) than stable encounters. Fig. 1. shows the normalized histograms of number of daily measurements for different features for stable and unstable encounters for patients who were in the unit for longer than 24 h. The histograms have been truncated to 24 measurements per day, although in a small proportion of the cohort, features were measured more than 24 times per day. The horizontal axis of the graph starts from 1 measurement per day and continues to 24 measurements per day.

**Fig. 2:**
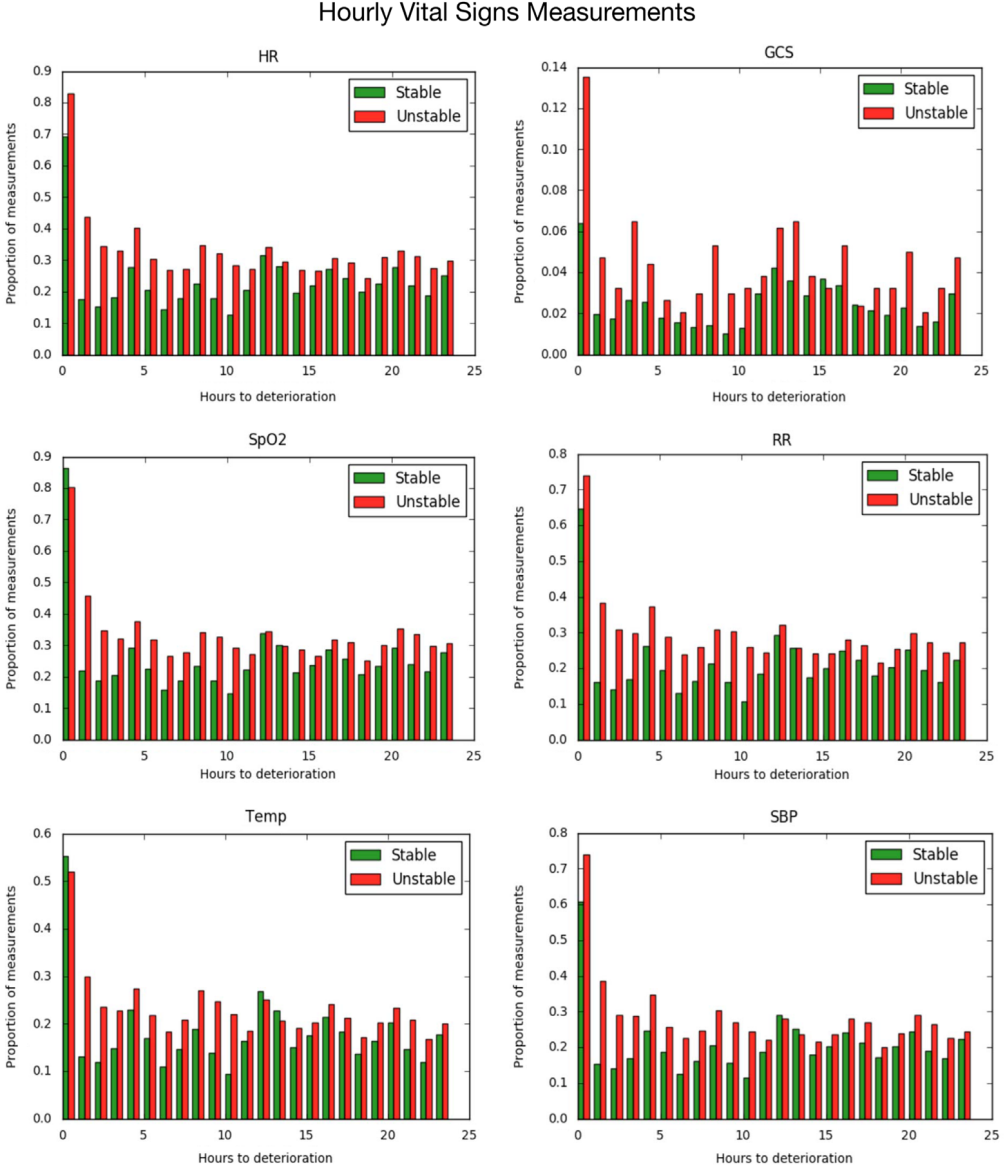
Graphs showing the proportion of stable (green) and unstable (red) cohort who had a measurement of the feature at each hour, for 24 h preceding deterioration/discharge.

In addition to analyzing the frequency of measurement we also computed the number of encounters (stable and unstable) a vital sign measured at each hour starting from the hour of discharge/transfer to higher level of care, going back 24 h, shown in Fig. 2. These figures show that as patients get closer to deterioration (red bars) a larger proportion of patients get the feature measurement compared to stable patients who are getting close to discharge (green bars).

## 2 Experimental design, materials and methods

Data was collected from electronic medical records for all patients admitted to medical/surgical unit (general ward) over a period of 2 years in a community hospital in Arizona, USA. Data collection was done in 2 phases- in the first phase data from 9265 patients (11864 encounters) was collected and in the second phase, data from 2097 patients (2418 encounters) was collected. Thus, data from a total of 11362 patients (14282 encounters) was collected. This included vital signs, information on admission and discharge times and information on location in the hospital (e.g. general ward, ICU etc.) More details are provided in Ghosh et al. 2017 [1].

Deterioration was defined as death or transfer to higher level of care such as progressive care unit (PCU) or ICU [2], [3], [4]. We classified episodes into “stable” and “unstable” classes based on their discharge location. We calculated the number of measurements taken for each patient in a 24 h period for 6 vital signs- HR, RR, SpO_2_, SBP, temperature and LOC. Patients were grouped by their outcome into stable and unstable as defined above. We graphed two histograms for each vital sign, one for each group of patients. These histograms were truncated to a maximum value of 24 measurements per day (1 measurement per hour). This is shown in Fig. 1.

We also calculated the number of patients with a selected vital sign measured at each hour starting from discharge/transfer to higher level of care to 24 h prior. For this analysis, patients who were in the general ward for longer than 24 h were selected (Fig. 2). Patients were grouped into stable & unstable as above.
