# Supplementary material for: Description of vital signs data measurement frequency in a medical/surgical unit at a community hospital in United States
Source: Data Brief. 2017 Nov 21;16:612–6. doi: 10.1016/j.dib.2017.11.053 (PMC5726752; doi:10.1016/j.dib.2017.11.053)
Supplement: Supplementary file 1 — Supplementary material [file mmc1.pdf]

PHILIPS

Philips Research

June 19, 2017

Conflict of Interest

This research was conducted by the authors as a part of their duties while employed at Philips Research North America. Erina Ghosh and Eric Carlson are currently employed at Philips Research North America. Larry Eshelman currently serves as a consultant to Philips Research. Lin Yang and Bill Lord were formerly employed at Philips Research North America.
All authors have read and agree to the above statements.

Yours sincerely,

Erina Ghosh, Ph.D.
Senior Scientist,
Acute Care Solutions
Email: Erina.ghosh@philips.com

Philips Research North America
Two Canal Park, 3rd Floor
Cambridge, MA 02141
Tel. (617)613-2200
www.research.philips.com
